# Does Patellar Height Influence Range of Motion and Anterior Knee Pain after Distal Femur Endoprosthesis Reconstruction?

**DOI:** 10.3390/jcm13144194

**Published:** 2024-07-18

**Authors:** Andrea Sambri, Chiara Paganelli, Stefania Claudia Parisi, Matteo Filippini, Luca Cevolani, Davide Stimolo, Marta Bortoli, Andrea Guarino, Alessandro Bruschi, Michele Fiore, Domenico Andrea Campanacci, Davide Maria Donati, Massimiliano De Paolis

**Affiliations:** 1 Orthopaedics and Traumatology Unit, IRCCS Azienda Ospedaliero-Universitaria di Bologna, 40138 Bologna, Italy; chiara.paganelli8@studio.unibo.it (C.P.); stefania.parisi@ior.it (S.C.P.); matteo.filippini@ior.it (M.F.); marta.bortoli@ior.it (M.B.); alessandro.bruschi@aosp.bo.it (A.B.); michele.fiore@aosp.bo.it (M.F.); massimiliano.depaolis@aosp.bo.it (M.D.P.); 2 Orthopaedic Oncology Unit, IRCCS Istituto Ortopedico Rizzoli, 40136 Bologna, Italy; luca.cevolani@ior.it (L.C.); davide.donati@ior.it (D.M.D.); 3 Orthopaedic Oncology Unit, Azienda Ospedaliera Universitaria Careggi, 50134 Firenze, Italy; davide.stimolo@unifi.it (D.S.); andrea.guarino@unifi.it (A.G.); domenico.campanacci@unifi.it (D.A.C.)

**Keywords:** distal femur, endoprosthetic replacement, patellofemoral, anterior knee pain, range of motion

## Abstract

**Objectives**: This study aims to evaluate the patellar height changes after distal femur (DF) endoprosthetic replacement (EPR) and its impact on anterior knee pain (AKP) and range of motion (ROM). **Methods:** A retrospective review of three institutions’ databases was performed. The patellar height was determined using the modified Insall–Salvati ratio (MIS), the Blackburne–Peel (BP) and the Caton–Deschamps (CD) indexes. Data regarding AKP and ROM were collected. **Results**: A total of 199 patients were included. The mean age at presentation was 37.9 ± 23.1 years. The mean one-year follow-up MIS, BP and CD were 1.52 (sd: 0.41), 0.82 (sd: 0.33) and 0.93 (sd: 0.33). Patellar height decreased significantly compared to the pre-operative values according to all three scores (*p* < 0.001). AKP was reported by 34 (17.1%) patients at 1 year follow-up. Patients with patella baja (MIS < 1.2) or pseudo patella baja (CD < 0.6) had a higher incidence of AKP (*p* = 0.037 and *p* = 0.024, respectively). The mean flexion ROM was 91°, with a direct correlation with patellar height (MIS *p* = 0.020, BP *p* = 0.036 and CD *p* = 0.036). **Conclusion**: The restoration of the native position of the joint line in DF EPR is important to maintain optimal patellofemoral biomechanics. Despite surgeons’ tendency toward a reduction in patellar height with respect to pre-operative values, an increase in patellar height might help to achieve better knee flexion and reduce AKP.

## 1. Introduction

Abnormalities in patella height are recognized complications of total knee arthroplasty (TKA) [1] and are thought to be associated with anterior knee pain (AKP) and decreased range of motion ROM) [2]. In particular, patella baja has been reported to result in inferior outcomes after TKA [3], whereas patella alta can cause maltracking, instability, and general dissatisfaction [4].

Several studies have linked an elevated joint line and the resultant patella baja to inferior clinical and functional results also in revision TKA [5,6,7]. Failure to restore the joint line has been shown to decrease the range of motion (ROM) and compromise clinical outcomes [8,9].

Distal femoral (DF) resection and endoprosthetic reconstruction (EPR) implanted after oncologic resections differ significantly from TKA performed to treat osteoarthritis. The endoprostheses used in the oncologic settings are constrained rotating hinge devices. The constrained hinge improves stability but entails less physiologic movement, thus potentially affecting the knee extensor mechanism. In addition, oncologic resections around the knee include the removal of an extensive amount of bone and soft tissue, increasing scar formation and potentially affecting patellar height. In particular, in distal femur EPR, the restoration of the native joint line is challenging, depending on the amount of tibial plateau resection, and there is a risk of overstuffing the knee in cases of excessive length of the femoral component. Therefore, it is difficult to define and maintain the correct patellar height in DF EPR after oncologic resections [10]. Moreover, restoring the native alignment could be challenging in the absence of anatomic landmarks; in these situations, there are limited techniques that guide surgeons intra-operatively to restore the rotation, such as the linea aspera, which is usually used as a landmark to identify the true posterior of the femur [11].

Change in patellar height has been studied for proximal tibia EPR. In these cases, over time, the patella gradually migrates proximally and affects knee performance [12,13]. Nevertheless, only a few studies have investigated the patellofemoral joint after DF tumor resection [10,14,15].

This study aims to evaluate the patellar height changes after DF EPR and its impact on AKP, ROM, and function.

## 2. Methods

A retrospective review of the prospectively maintained databases of three institutions was undertaken for all patients treated with DF resection for oncologic reasons and EPR between 2010 and 2022. 

The study was conducted in accordance with the Declaration of Helsinki and approved by the Institutional Review Board (n° 793/2021/Oss/AOUBo). Only patients with at least 12 months of follow-up and patients who provided informed consent were included. Patients with EPR failure before 12 months of follow-up were excluded. We also excluded patients who underwent extra-articular knee resection, revision, patellectomy, reconstruction with an expandable prosthesis, or proximal tibial replacement associated with the DF EPR. We also excluded patients when imaging and/or clinical data were incomplete.

**Figure 1:**
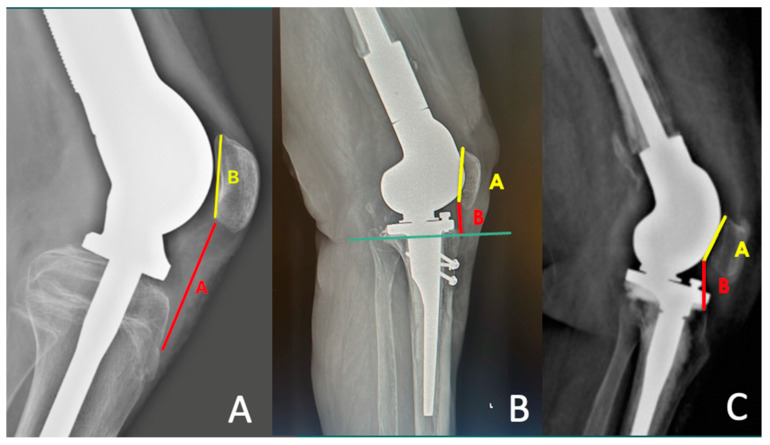
(**A**) modified Insall–Salvati ratio (MIS) = A/B: A: distance from the inferior margin of the patellar articular surface to the patellar tendon insertion, B: length of the patellar articular surface; (**B**) the Blackburne–Peel (BP) index = B/A: horizontal line at the level of the tibial plateau is drawn. A: line along the patellar articular surface, B: distance between the horizontal line and the inferior aspect of the patellar articular surface; (**C**) the Caton–Deschamps (CD) index = A/B: A: patellar articular surface length, B: distance between the anterior angle of the tibial plateau, to the most inferior aspect of the patellar articular surface.

The patellar height was determined by the modified Insall–Salvati ratio (MIS) [16], the Blackburne–Peel (BP) index [17] and the Caton–Deschamps (CD) index [18] on lateral radiographs taken at 30° of flexion. [19,20] (Figure 1).

The measurements were verified by two surgeons (SC.P. and Ma.F.) using digital radiography [21] and checked by the senior author (M.DP.) All measures were collected pre-operatively and one year after surgery.

The tibial plateau–fibula apex distance (TPFA) was measured as the distance between the line perpendicular to the apex of the fibular head and the parallel line to the bone interface of the tibial plate.

The restoration of the length of the resected femur was estimated as the difference between the length of the distal femur specimen and the length of the DF EPR.

Data regarding anterior knee pain (AKP) and range of motion were collected at one-year follow-up.

Anterior knee pain was graded according to the criteria of Waters and Bentley [22], with Grade 0 indicating no pain, Grade I indicating mild pain (does not interfere with daily activities), Grade II indicating moderate pain (patient not considering additional surgery), and Grade III indicating severe pain (patient considering additional surgery). However, to facilitate statistical analysis, only the presence or absence of pain was considered.

Patients were clinically assessed at one-year follow-up using the Knee Society score (KSS). KSS is a clinical rating system and is divided into two domains (knee score (KSS-K) and function score (KSS-F)) [23].

Quantitative data were summarized by frequencies and percentages for categorical variables, means, standard deviations and range for continuous variables.

The Shapiro–Wilk test was used to verify normal distribution and the Levene test was used to analyze homogeneity of the variances. A parametric test was used to compare samples in case of continuous variables and normal distribution. As a parametric test, Student’s two-tailed t-test was used to compare the average of the variables for homoscedastic unpaired groups, and the Welch t-test was used to compare non-homoscedastic unpaired groups. The two-tailed Mann–Whitney U test was used as non-parametric test for unpaired groups. Continuity correction was applied in the case of discrete distribution. Odds ratios were used to quantify the strength of the association between categorical variables using Pearson’s χ^2^ to establish significance. Pearson or Spearman coefficients were used to make correlations, depending on the adequacy of the variables analyzed. A *p*-value of < 0.05 was considered statistically significant. All analysis was completed using the Statistical Package for Social Science (IBM Corp. Released 2013. IBM SPSS Statistics for Windows, Version 26.0. IBM Corp.: Armonk, NY, USA). Graphs were obtained using GraphPad Prism 10 (GraphPad Software, San Diego, CA, USA).

## 3. Results

During the study period, 325 DF EPR were performed at our institutions.

**Figure 2:**
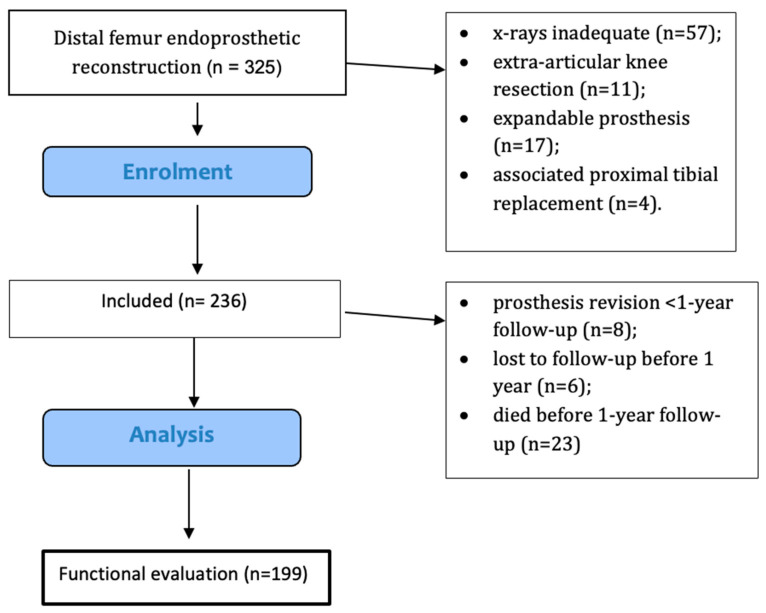
Flow diagram showing the included patients.

A total of 106 patients were excluded: 57 did not have adequate x-rays for analysis, 11 underwent an extra-articular knee resection, 8 had prosthesis revision before 1 year follow-up, 6 were lost to follow-up before 1 year, 23 died before 1 year follow-up; 17 expandable prosthesis and 4 associated proximal tibial replacements were also excluded. After these exclusions, the final cohort comprised 199 patients (Figure 2).

The mean age at presentation was 37.9 ± 23.1 years. The cohort included 119 (59.8%) male and 80 (40.2%) female patients.

The procedures were performed to treat 162 primary malignant bone tumors, 22 metastases and 15 aggressive benign bone tumors. The mean length of DF resection was 157 mm (range, 70–300). In 37 (18.6%) patients, radiotherapy was administered in a neoadjuvant setting. None of the patients received adjuvant radiotherapy.

All the implants used were rotating hinge and most of them were Megasystem-C, in 86 cases, (Waldemar Link GmbH & Co. KG, Hamburg, Germany) and GMRS (Stryker corp., Kalamazo, MI, USA), in 73 cases. Other implants included STANMORE^®^ modular megaprostheses (Stanmore Implants Worldwide Ltd., Middlesex, UK) in 19 cases, Howmedica Modular Resection System (HMRS) Howmedia Inc., Rutherford, NJ, USA) in 13 and Zimmer Segmental System (ZSS) (Zimmer Inc., Warsaw, IN, USA) in 8 cases.

All patients had non-resurfaced patellas.

The mean follow-up was 38 (range, 12–94) months.

The mean pre-operative MIS, BP and CD were 1.66 (sd: 0.39), 0.86 (sd: 0.29) and 1.01 (sd: 0.41), respectively.

**Figure 3:**
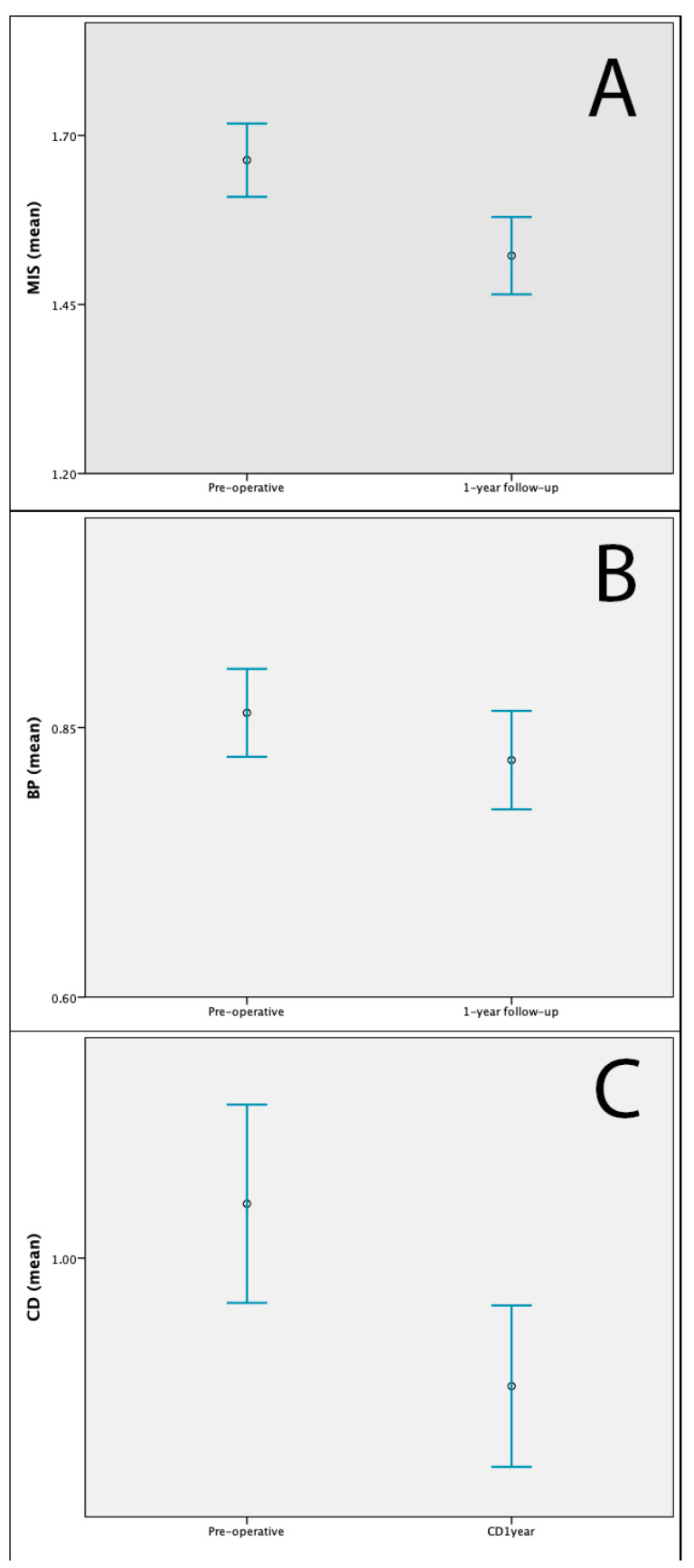
(**A**) Mean and 95% confidence intervals of pre-operative and 1 year follow-up MIS. (**B**). Mean and 95% confidence intervals of pre-operative and 1 year follow-up BP. (**C**). Mean and 95% confidence intervals of pre-operative and 1 year follow-up CD. MIS: modified Insall–Salvati ratio; BP: Blackburne–Peel index; CD: Caton–Deschamps (CD) index.

The mean one-year follow-up MIS, BP and CD were 1.52 (sd: 0.41), 0.82 (sd: 0.33) and 0.93 (sd: 0.33). Patellar height decreased significantly compared to the pre-operative values according to all three scores (*p* < 0.001, *p* < 0.001 and *p* < 0.001, respectively) (Figure 3).

The mean TPFA distance at one-year follow-up was 6.01 mm (range, 4.70–18.62).

We observed an inverse correlation between CD and TPFA (*p* = 0.047); a direct correlation between the length of resection and patellar height was found (MIS (*p* = 0.012), BP (*p* = 0.004) and CD (*p* = 0.004)).

According to CD, at the one-year follow-up, a total of 36 (18.1%) patients presented with pseudo patella baja (CD < 0.6), The remaining patients presented patella norma (149, 74.9%) or alta (CD > 1.3) (14, 7.0%).

After surgery, the distal femur was shortened by 8 mm on average (range, −18–+8).

Anterior knee pain was reported by 34 (17.1%) patients at 1 year follow-up. However, only four of them (11.8%) with grade III AKP required patellar resurfacing thereafter. Patients with patella baja (MIS < 1.2) or pseudo patella baja (CD < 0.6) had a higher incidence of AKP (*p* = 0.037 and *p* = 0.024, respectively), see Table 1.

Ten (5.0%) patients had extension lag at 1 year follow-up and were therefore excluded from ROM analysis. The mean flexion ROM in the remaining 189 cases was 91° (ranging from 20° to 110°). A direct correlation between ROM and patellar height was found (MIS *p* = 0.020, BP *p* = 0.036 and CD *p* = 0.036).

Functional evaluation at 1 year follow-up measured mean KSS-K 75.0 (sd: 18.8) and mean KSS-F 79.8 (sd: 19.3).

No significant correlation was found either between KSS-K and patellar height (MIS *p* = 0.074, BP 0.082, CD 0.052), or between KSS-F and patellar height (MSI *p* = 0.653, BP *p* = 0.882, CD *p* = 0.613).

## 4. Discussion

To the best of our knowledge, even though three previous series described patellar complications after DF EPR [10,15,24], only one directly focused on the effects of patellar height in DF EPR [24].

This study contributes to demonstrating that patellar height decreased significantly compared to pre-operative values, as previously reported by Etchebehere et al. [24]. Patella baja can be detected from the MIS ratio, whereas pseudo patella baja (caused by the elevation of the joint line) can be detected more reliably from the BP or CD ratios. Thus, the reduction in all indexes observed in the present study highlights that the decrease in patellar height was probably caused both by patellar tendon shortening and elevation of the articular surface [25]. One of the possible causes of patellar tendon shortening is post-surgical scarring which is attributed to the extensive soft tissue dissection in oncologic resections [2,10]. This might be mitigated by an early post-operative rehabilitation, even during chemotherapy, with supervised active and passive ROM and gait training starting the day after surgery [26]. On the other hand, pseudo patella baja can be produced by an alteration on both the tibial and femoral sides. The width of the tibial component in most EPR is approximately 12 mm, which makes it necessary to cut approximately this width of the tibial plateau to restore the tibial joint line. Moreover, surgeons might choose to reconstruct the distal femur slightly shorter than the resected specimen in order to avoid knee-joint overstuffing [27].

Another important finding of this study is that patients with patella baja/pseudo patella baja had a higher incidence of anterior knee pain. The incidence of AKP in our entire cohort was lower than that reported by Etchebehere et al. (24%) [15] and Schwab et al. (32%) [10]. However, we collected the data from clinical notes, thus potentially underestimating the prevalence of AKP. Differently from Etchebehere et al. [15], AKP was graded in our series. Only four patients with grade III AKP required patellar resurfacing. The reduced perception of symptoms could be related to the megaprosthesis design and to the limited flexion inherent to that type of reconstruction, which could ultimately make the pain more tolerable than patients with standard TKA [28].

The mean flexion ROM was 91°, almost equal to that reported by Schwab et al. [10]. This ROM enables the patients to perform most of their daily life activities. Our linear regression model showed a direct correlation between ROM and patellar height, similar to Schwab et al. [10]. These results corroborate with previous studies on TKA [2,29] that showed a correlation between patella baja and modification of the patellofemoral contact forces responsible for decreasing ROM.

Our study found only a tendency toward higher KSS-K scores at 1 year follow-up as the patellar height increased. This scale is mainly determined by pain and ROM. However, no correlation was found with functional KSS.

The main limitations of our study are related to its retrospective and multicentric nature; thus, it potentially suffers from the risk of selection bias. In particular, the lack of details about the grade of pre-operative patellar arthritis and radiographic data on the rotation of the EPR might have influenced our observations. Also, the small numbers in each subgroup did not allow any further analysis. Moreover, in such a retrospective study it was not possible to collect reliable data on the pre-operative duration of symptoms, prosthesis rotation measurement, limb length discrepancy, and post-operative physiotherapy. All these factors might contribute to post-operative AKP. The size of the distal femur EPR might also influence patellar tracking and AKP.

However, differently from previous similar series, the follow-up data were homogenized at one-year follow-up. The strength of this study is the number of patients included, and the comprehensive statistical analysis. Moreover, the measure of different patellar height indexes might add supplementary information. Nevertheless, cut-off points for patella baja/pseudo patella baja were adopted from standard TKA, as they have not yet been defined for EPR.

Samargandi et al. [11] already highlighted the importance of restoring length and rotation after DF resections, proposing the use of an intra-operative external fixator to maintain length and rotation of the prosthesis. Our results confirm that the position of the joint line deserves special attention in a DF EPR. It is desirable to restore the native position of the joint line to maintain optimal patellofemoral biomechanics. Careful measurement of the resected specimen and the tibial plateau resection are necessary steps. The planning of the length of the prosthetic reconstruction must include the length of the tibial tray and polyethylene in addition to the distal femoral prosthesis. Despite surgeons’ tendency toward a reduction in patellar height with respect to pre-operative values, an increase in patellar height might help to achieve better flexion of the knee and reduce anterior knee pain.

## Figures and Tables

**Table 1:** Characteristics at 1 year follow-up in patients with and without anterior knee pain.

	No Pain (*n* = 165)	Anterior Knee Pain (*n* = 34)	*p* *
TPFA 1 year follow-up (mean, range)	6.23 (−4.70–18.11)	4.84 (−5.40–18.62)	0.281
MIS 1 year follow-up (mean, range)	1.51 (0.48–3.56)	1.56 (1.02–2.82)	0.470
BP 1 year follow-up (mean, range)	0.81 (0.34–1.62)	0.86 (0.38–1.45)	0.560
CD 1 year follow-up (mean, range)	0.92 (0.24–1.64)	0.96 (0.43–1.98)	0.878
Extension lag (*n*=)	6	2	0.243
Flexion ROM, ° (mean, range)	91.7° (20°–130°)	87.1° (20°120°)	0.235
KSS-K 1 year follow-up (mean, range)	77.5 (19–100)	62.4 (30–93)	<0.001
KSS-F 1 year follow-up (mean, range)	81.4 (25–100)	72.2 (25–100)	0.001
Anterior knee pain grading			
Grade 0	165	0	
Grade I	0	22	
Grade II	0	8	
Grade III	0	4	

MIS: Modified Insall–Salvati ratio; BP: Blackburne–Peel (BP) index CD: Cadon–Dechamps index; KSS-K: Knee Society knee score; KSS-F: Knee Society function score (KSS-F) [23]. TPFA: tibial plateau–fibula apex distance. * univariate anova.

## Data Availability

Data would be available upon request to the corresponding author.
